# The loss of the kinases SadA and SadB results in early neuronal apoptosis and a reduced number of progenitors

**DOI:** 10.1371/journal.pone.0196698

**Published:** 2018-04-26

**Authors:** Pratibha Dhumale, Sindhu Menon, Joanna Chiang, Andreas W. Püschel

**Affiliations:** 1 Institut für Molekulare Zellbiologie, Westfälische Wilhelms-Universität, Münster, Germany; 2 Cells-in-Motion Cluster of Excellence, University of Münster, Münster, Germany; Osaka University, JAPAN

## Abstract

The neurons that form the mammalian neocortex originate from progenitor cells in the ventricular (VZ) and subventricular zone (SVZ). Newborn neurons are multipolar but become bipolar during their migration from the germinal layers to the cortical plate (CP) by forming a leading process and an axon that extends in the intermediate zone (IZ). Once they settle in the CP, neurons assume a highly polarized morphology with a single axon and multiple dendrites. The AMPK-related kinases SadA and SadB are intrinsic factors that are essential for axon formation during neuronal development downstream of Lkb1. The knockout of both genes encoding Sad kinases (*Sada* and *Sadb*) results not only in a loss of axons but also a decrease in the size of the cortical plate. The defect in axon formation has been linked to a function of Sad kinases in the regulation of microtubule binding proteins. However, the causes for the reduced size of the cortical plate in the *Sada*^*-/-*^*;Sadb*^*-/-*^ knockout remain to be analyzed in detail. Here we show that neuronal cell death is increased and the number of neural progenitors is decreased in the *Sada*^*-/-*^*;Sadb*^*-/-*^ CP. The reduced number of progenitors is a non-cell autonomous defect since they do not express Sad kinases. These defects are restricted to the neocortex while the hippocampus remains unaffected.

## Introduction

The six layers of the mammalian neocortex are formed by successive waves of neurons that are generated by apical progenitor cells (APCs) located in the VZ and intermediate progenitor cells (IPCs) in the SVZ [1, 2]. Newborn neurons initially have a multipolar morphology before they become polarized by forming an axon and a leading process [3–6]. After assuming a bipolar morphology neurons migrate along the radial glia cells (RGCs) into the CP while the axon rapidly extends in the IZ. A similar process to establish neuronal polarity can be observed in cultures of neurons from the embryonic brain [5, 7]. Initially, unpolarized neurons extend several undifferentiated neurites (stage 2 of polarization) in culture. These multipolar neurons polarize by selecting one of the neurites as the axon that begins to extend rapidly (stage 3).

Many intrinsic and extrinsic factors have been identified that regulate the polarization of neurons [5–7]. One of these pathways includes the kinases SadA and SadB (also known as Brsk2 and Brsk1) [8]. The evolutionarily conserved Sad kinases were first identified in *C*. *elegans* where the phenotype of Sad-1 mutants revealed a function in neuronal polarity and synapse development [9, 10]. Sad-1 localizes to synapse-rich regions of axons and regulates several aspects of presynaptic differentiation. The mammalian SadA and SadB show a high sequence similarity to each other and to Sad-1 with the highest homology in their kinase domain [8]. SadA and SadB belong to a large family of kinases with 13 members in mammals that are related to the adenosine 5'-monophosphate (AMP)-activated protein kinases (AMPKs) [11, 12]. Like the other AMPK-related kinases SadA and SadB are regulated by the LKB1/STRAD complex that activates them by phosphorylating Thr175 and Thr187, respectively.

SadA and SadB are required for the formation of axons in the cortex [8, 13]. Outside the cortex they regulate the central arborization of a subset of sensory axons [14, 15]. In addition, they are involved in the structural and functional maturation of synapses in both the central and peripheral nervous systems [14, 15]. A knockout of both *Sada* and *Sadb* shows a severe reduction in axon tracts in the cortical IZ and a thinning of the cortex at E17, which is not detectable in the single knockouts [8]. The cortical phenotype of *Sada*^*-/-*^*;Sadb*^*-/-*^ mutants resembles that of cortex-specific *Lkb1* and *Strada*^*-/-*^*;Stradb*^*-/-*^ knockouts [13, 16]. Cultured hippocampal neurons from the brain of *Sada*^*-/-*^*;Sadb*^*-/-*^ double knockout embryos fail to form distinct axons or dendritic processes [8, 17]. Instead, they extend multiple long neurites, which are positive for both axonal and dendritic markers. The defect in the formation of axons has been attributed to a function of Sad kinases in the regulation of microtubule binding proteins [8, 13]. However, it has not been analyzed how the decrease in the size of the cortex develops in the *Sada*^*-/-*^*;Sadb*^*-/-*^ double mutant.

Here we analyze the phenotype of *Sada*^*-/-*^*;Sadb*^*-/-*^ double knockout mice between embryonic day 13 (E13) and E17. We show that the reduced size of the cortex is linked to an extensive apoptosis of neurons in the CP beginning at E15 and a reduction in the number progenitors. The expression of Sad kinases is restricted to neurons and could not be detected in progenitors, indicating that the reduced number of progenitor cells is a cell non-autonomous defect.

## Results

### Defects in cortical development in *Sada*^*-/-*^*;Sadb*^*-/-*^ knockout mice

**Fig 1:**
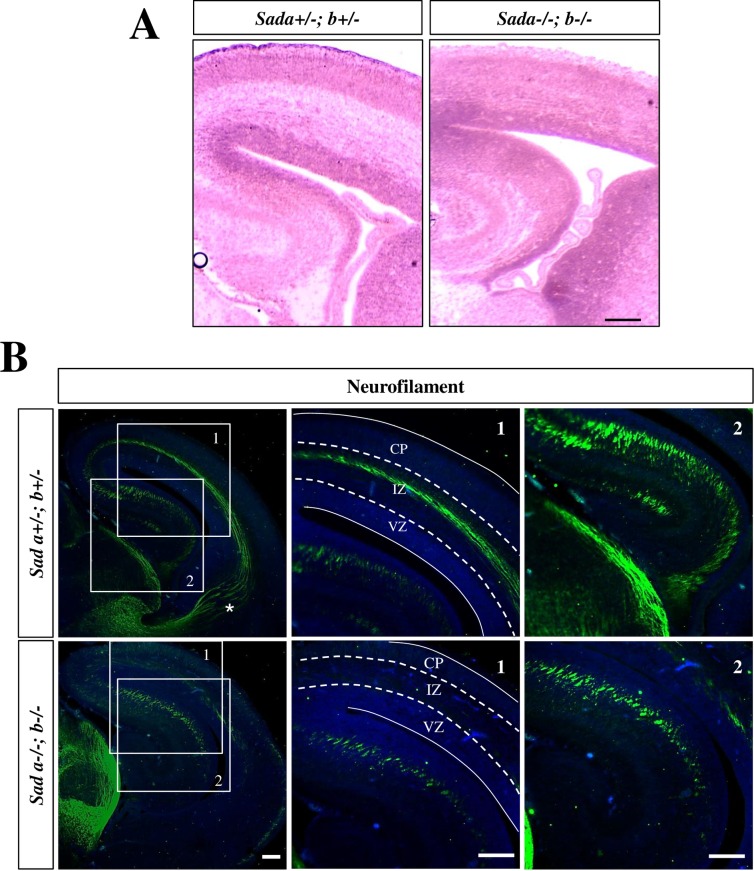
Reduced cortical size and loss of axons in *Sada*^*-/-*^*;Sadb*^*-/-*^ embryos. Coronal sections from the brains of *Sada*^*+/-*^*;Sadb*^*+*/-^ and *Sada*^*-/-*^*;Sadb*^*-/-*^ E17 embryos were analyzed by (A) Hematoxylin-Eosin staining or (B) staining with Hoechst 33342 (blue) and an anti-NF-M antibody (2H3) to mark the axons in the IZ of the cortex and the hippocampus. The boundaries between VZ/SVZ, IZ and CP are indicated by broken lines. The thalamocortical projection (marked by an asterisks) is missing in the *Sada*^*-/-*^*;Sadb*^*-/-*^ cortex. The scale bars are 100 μm (A, B) and 50 μm (insets, B), respectively.

The knockout of both genes encoding Sad kinases has a severe effect on cortical development (Fig 1A; S1A Fig). The radial diameter of the cortex is reduced by 37% from 324 ± 8 μm to 204 ± 11 μm (p<0.001) and the ventricles are expanded in *Sada*^*-/-*^*;Sadb*^*-/-*^ embryos at E17 compared to heterozygous *Sada*^*+/-*^*;Sadb*^*+*/-^ controls as previously reported [8]. In addition, the organization of the cortex into distinct CP and IZ is lost while the VZ and SVZ were still distinguishable. To define the time point when these defects can be first observed, we analyzed earlier stages of development using neurofilament medium chain (NF-M) as marker. NF-M immunoreactivity was detectable in the most superficially located neurons at the top of the CP in the *Sada*^*-/-*^*;Sadb*^*-/-*^ knockout similar to heterozygous *Sada*^*+/-*^*;Sadb*^*+*/-^ controls at E13 and E14 (S1B Fig). Differences started to become apparent at E15 when NF-M^+^ neurons were distributed over a larger area of the *Sada*^*-/-*^*;Sadb*^*-/-*^ knockout cortex compared to the *Sada*^*+/-*^*;Sadb*^*+*/-^ brain.

The cortex of E17 *Sada*^*-/-*^*;Sadb*^*-/-*^ knockout embryos was almost completely devoid of signals for the axonal marker NF-M at E17 (Fig 1B). In addition, the thalamocortical projection was missing but it remains to be analyzed whether this results from an intrinsic defect in axon formation or a guidance defect due the loss of their interaction with cortical axons [18, 19]. By contrast, NF-M staining was still present in the hippocampus (Fig 1B). This difference in the phenotype with respect to in axon formation was also observed in cultures of cortical and hippocampal neurons prepared from the brains of E17 embryos (S2 Fig). Cortical neurons from the *Sada*^*-/-*^*;Sadb*^*-/-*^ knockout brain extended several short neurites at 3 d.i.v. that were positive for MAP2 while NF-M staining was completely absent (S2A Fig). By contrast, hippocampal *Sada*^*-/-*^*;Sadb*^*-/-*^ knockout neurons formed multiple neurites, which are uniform in length and positive for both axonal (NF-M and Tau-1) and dendritic markers (MAP2) at 3 d.i.v. (S2B and S2D Fig). Thus, the loss of Sad kinases results in in the complete loss of axons in cultured cortical neurons while hippocampal neurons extend multiple neurites that have characteristics of both axons and dendrites.

### Loss of neurons in the *Sada*^*-/-*^*;Sadb*^*-/-*^ cortex but not hippocampus

**Fig 2:**
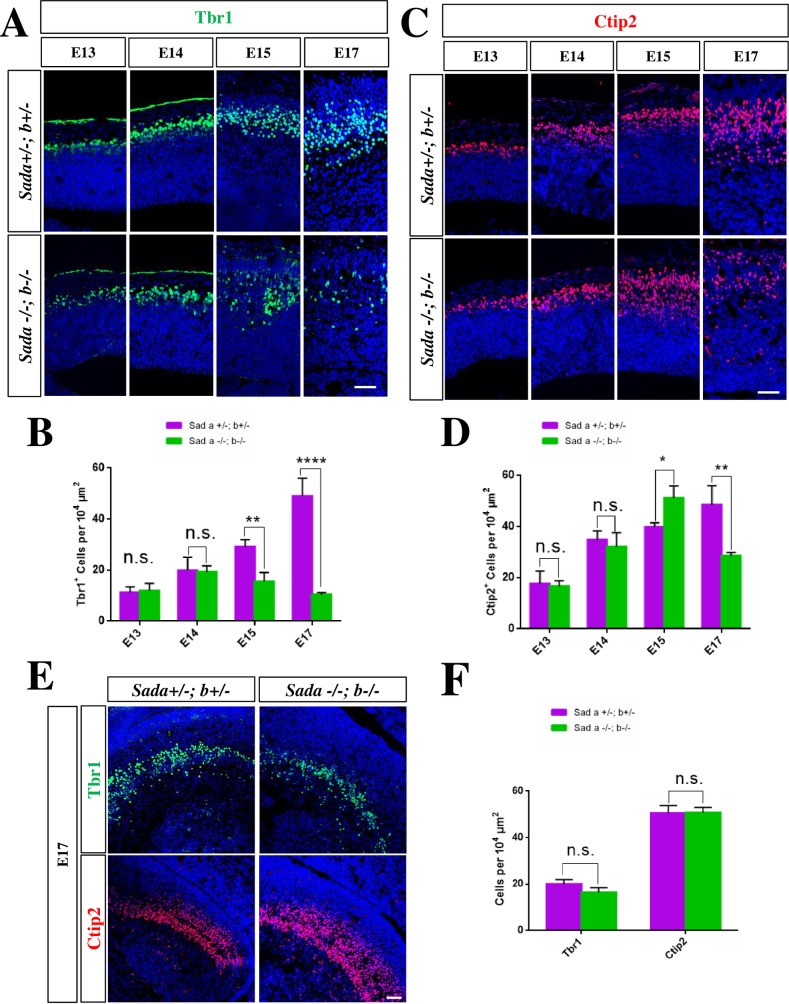
A loss of neurons in the *Sada*^*-/-*^*;Sadb*^*-/-*^ cortex is first observed at E15. (A, C, E) Coronal sections from the brains of E13 to E17 embryos with the indicated genotype were stained with antibodies for Tbr1^+^ (marker for L5/6 and subplate; green) or Ctip2^+^ (L4–6; red) and Hoechst 33342 (blue). (B, D, F) The number of Tbr1^+^ (B, F) and Ctip2^+^ cells (D, F) in the cortex (B, D) and hippocampus (F, E17) normalized to an area of 10^4^ μm^2^ is shown for the *Sada*^*+/-*^*;Sadb*^*+*/-^ (magenta) and *Sada*^*-/-*^*;Sadb*^*-*/-^ cortex (green). Values are means ± s.e.m., n = 3 different embryos, two-way ANOVA compared to heterozygous control; *, p<0.05; **, p<0.01, ***, p<0.001. The scale bars are 50 μm.

To determine if the formation of cortical layers is affected in the *Sada*^*-/-*^*;Sadb*^*-/-*^ knockout cortex we used the layer-specific markers Tbr1 (subplate and deeper layer 5 (L5) and L6) and Ctip2 (L4 to L6). The number and distribution of Tbr1^+^ or Ctip2^+^ neurons was similar in the *Sada*^*-/-*^*;Sadb*^*-/-*^ knockout brain and heterozygous *Sada*^*+/-*^*;Sadb*^*+*/-^ controls at E13 and E14 (Fig 2A–2D, Table 1). By E15, however, the number of Tbr1^+^ neurons was reduced from 29 ± 2 cells per 10^4^ μm^2^ in controls to 16 ± 2 cells in the *Sada*^*-/-*^*;Sadb*^*-/-*^ double knockout cortex. While the loss of Tbr1^+^ neurons is detectable at E15 and E17, there was a transient increase of Ctip2^+^ neurons at E15 (Fig 2D, Table 1) before their number also declined at E17. A dramatic reduction in the number of both Tbr1^+^ (heterozygous control: 49 ± 4 cells per 10^4^ μm^2^) and Ctip2^+^ neurons (49 ± 4 cells per 10^4^ μm^2^) to 10 ± 2 and 28 ± 1 cells per 10^4^ μm^2^, respectively, was observed in the *Sada*^*-/-*^*;Sadb*^*-/-*^ double knockout cortex at E17 (Fig 2A–2D; Table 1). The Tbr1^+^ and Ctip2^+^ neurons did not form a well-organized layer and were present at deeper positions than normal beginning at E15 (Fig 2A and 2C). By contrast, a similar loss was not observed in the hippocampus (Fig 2E and 2F). These results show that the formation of cortical layers is disturbed beginning at E15 in the absence of Sad kinases.

**Table 1:** Defects in cortical development in the *Sada*^-/-^;*Sadb*^-/-^ knockout.

	Stage	*Sada*^*+/-*^*; Sadb*^*+/-*^	*Sad* ^*-/-*^*; Sadb*^*-/-*^
**Tbr1^+^ cells per 10^4^ μm^2^**	**E13**	11 ± 1	12 ± 2
	**E14**	20 ± 3	19 ± 1
	**E15**	29 ± 2	16 ± 2
	**E17**	49 ± 4	10 ± 1
**Ctip2^+^ cells per 10^4^ μm^2^**	**E13**	18 ± 2	16 ± 1
	**E14**	35 ± 2	32 ± 3
	**E15**	39 ± 1	51 ± 2
	**E17**	49 ± 4	28 ± 1
**Pax6^+^ cells per 10^4^ μm^2^**	**E13**	128 ± 16	130 ± 12
	**E15**	102 ± 12	54 ± 4
	**E17**	102 ± 9	58 ± 5
**Tbr2^+^ cells per 10^4^ μm^2^**	**E13**	59 ± 3	58 ± 2
	**E15**	69 ± 1	52 ± 1
	**E17**	66 ± 2	41 ± 2
**Ki67^+^ cells per 10^4^ μm^2^**	**E13**	85 ± 3	84 ± 3
	**E15**	72 ±2	57 ± 5
	**E17**	72 ± 3	41 ± 6
**PH3^+^ cells per section**	**E13**	89 ± 8	93 ± 6
	**E15**	70 ± 4	72 ± 2
	**E17**	65 ± 4	61 ± 4
**PCNA^+^ cells with punctate staining per 10^4^ μm^2^**	**E13**	23 ± 2	22 ± 2
	**E15**	11 ± 2	8 ± 1
	**E17**	8 ± 1	2 ± 1
**mitotic index** (ratio of PH3^+^ and Ki67^+^cells in %)	**E13**	9 ± 1	9 ± 1
	**E15**	6 ± 1	8 ± 2
	**E17**	6 ± 1	11 ± 2
**proportion of S-phase cells** (ratio of PCNA^+^ S-phase and Ki67^+^cells in %)	**E13**	25 ± 3	25 ± 3
	**E15**	20 ± 3	18 ± 2
	**E17**	11 ± 3	12 ± 3
**CC3^+^ cells per section**	**E15**	N.D.	68 ± 14
	**E17**	N.D.	144 ± 12
**phospho-Ser139-γH2A.X^+^ cells per section**	**E15**	N.D.	9 ± 2
	**E17**	N.D.	21 ± 3

The number of cells positive for the analyzed markers was determined in the CP (Ctip2, Tbr1) or VZ/SVZ (Ki67, Pax6, PCNA, PH3, Tbr2) and normalized as the number of cells per 10^4^ μm^2^. To determine the mitotic index as the ratio of PH3^+^ and Ki67^+^cells the total number of PH3^+^ and Ki67^+^ cells per section was counted in the cortex. The number of S-phase cells was determined as the number of cells with a punctate PCNA staining in the nucleus. Values are means ± s.e.m., N.D., not detectable.

### Reduced number of neural progenitors in the *Sada*^*-/-*^*;Sadb*^*-/-*^ cortex

**Fig 3:**
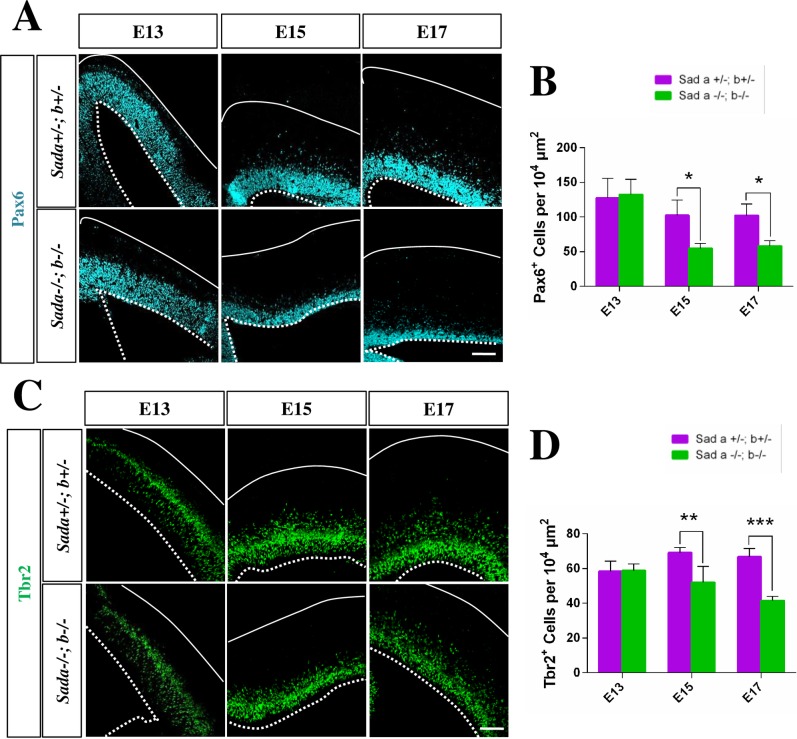
The loss of Sad kinases leads to a decrease in the number of neuronal progenitors in the cortex. (A-D) Coronal sections from the brains of E13, E15 or E17 embryos with the indicated genotypes were analyzed by staining with anti-Pax6 (APCs, A, pseudo-colored cyan), anti-Tbr2 (IPCs, B, green). Nuclei were stained with Hoechst 33342 (blue). The number of Pax6^+^ (B), Tbr2^+^ (D) cells per 10^4^ μm^2^ area in ventricular surface was quantified in the *Sada*^*+/-*^*;Sadb*^*+*/-^ (magenta) and *Sada*^*-/-*^*;Sadb*^*-*/-^ cortex (green). Values are means ± s.e.m., n = 3 different embryos, two-way ANOVA compared to heterozygous control; *, p<0.05; **, p<0.01; ****, p<0.0001). The Scale bars are 50 μm.

The number of neurons and the size of the cortex are determined by the balance of symmetric and asymmetric divisions of neural progenitors [20]. A quantification of the number of Pax6^+^ APCs and Tbr2^+^ IPCs [21, 22] did not reveal a significant difference between the *Sada*^*-/-*^*;Sadb*^*-/-*^ knockout cortex and heterozygous controls at E13 (Fig 3A–3D, Table 1). A significant decrease in the number of both Pax6^+^ and Tbr2^+^ cells was observed at E15 when also the first defects in the formation of cortical layers were apparent (Fig 3A–3D, Table 1). This decrease became more pronounced at E17. The loss of progenitors was not caused by a structural defect of RGCs. Staining with an anti-nestin antibody as a marker for RGCs did not reveal a defect in their organization in the *Sada*^*-/-*^*;Sadb*^*-/-*^ knockout brain at E13 or E17 (S3 Fig).

**Fig 4:**
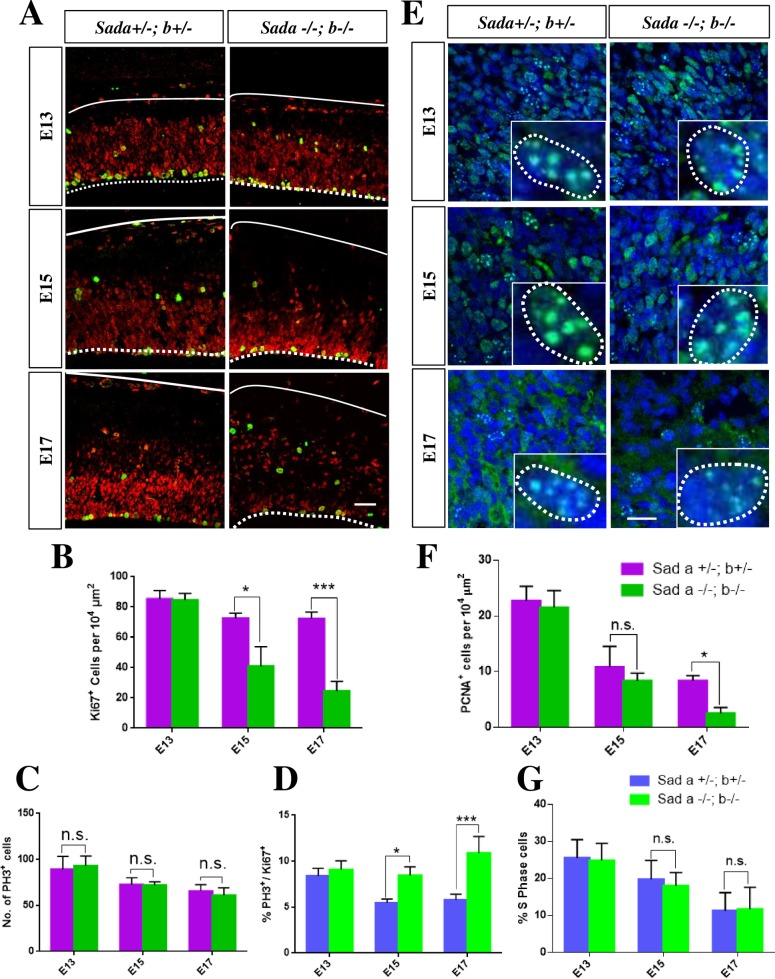
The loss of Sad kinases leads to an increase in the mitotic index. (A, E) Coronal sections from the brains of E13, E15 or E17 embryos with the indicated genotypes were analyzed by staining with anti-Ki67 (A, proliferating cells, red) and anti-PH3 antibodies (A, cells in M-phase, green) or with an anti-PCNA antibody (E, green) and Hoechst 33342 (blue). (B—D) A significant decrease in the number of proliferating Ki67^+^ cells per 10^4^ μm^2^ (B) was observed at E15 and E17 when comparing the *Sada*^*+/-*^*;Sadb*^*+*/-^ (magenta (B, C) and blue (D), respectively) and *Sada*^*-/-*^*;Sadb*^*-*/-^ cortex (green). The number of mitotic PH3^+^ cells per section remained constant (C) but the mitotic index (D, ratio of PH3^+^ and Ki67^+^cells in %) increased in the *Sada*^*-/-*^*;Sadb*^*-*/-^ cortex (green) at E15 and E17 compared to *Sada*^*+/-*^*;Sadb*^*+*/-^controls since the number of Ki67^+^cells was reduced. (F—G) The number of PCNA^+^ cells in S-phase (PCNA^+^ cells with a punctate staining pattern (inset)) per 10^4^ μm^2^ (F) and the proportion of cells in S-phase (G, ratio of PCNA^+^ S-phase and Ki67^+^cells in %) in the VZ/SVZ of the cortex were quantified in the *Sada*^*-/-*^*;Sadb*^*-*/-^ cortex (green) and *Sada*^*+/-*^*;Sadb*^*+*/-^controls (magenta (F) and blue (G), respectively). Values are means ± s.e.m., n = 3 different embryos, two-way ANOVA compared to heterozygous control; *, p<0.05; **, p<0.01; ***, p<0.001, n. s., not significant). The Scale bars are 20 μm.

To examine the cause for the reduced number of neural progenitors we stained sections with markers for proliferating (Ki67) and mitotic cells (phospho-histone 3 (PH3)). Consistent with the reduction in the number of Pax6^+^ and Tbr2^+^ cells, a significant decrease in the number of proliferating Ki67^+^ cells was observed in the *Sada*^*-/-*^*;Sadb*^*-/-*^ knockout cortex at E15 and E17 (Fig 4A and 4B, Table 1). The number of mitotic cells was comparable in the *Sada*^*-/-*^*;Sadb*^*-/-*^ knockout cortex and controls at all stages (Fig 4C, Table 1). A quantification of the number of cells that are positive for both Ki67 and PH3 (Fig 4A–4D) showed a significant increase in the mitotic index (percentage of Ki67^+^ cells that are PH3^+^) in the *Sada*^*-/-*^*;Sadb*^*-/-*^ knockout cortex at E15 and E17 (*Sada*^*-/-*^*;Sadb*^*-/-*^: 9 + 1% at E15, 11 ± 1% at E17; heterozygous control: 6 + 1% at E15, 6 ± 1% at E17) but not at E13 (Fig 4D, Table 1). To determine the proportion of cells in S-phase we quantified the number of cells with a punctate PCNA staining pattern that specifically marks cells in S-phase (Fig 4E) [23]. No difference was observed compared to controls at any stage of development (Fig 4E and 4F) (Fig 4E, 4F and 4G, Table 1). These results show that the number of apical and intermediate progenitors is decreased and the proportion of proliferating cells that are in M-phase is increased beginning at E15 in the *Sada*^*-/-*^*;Sadb*^*-/-*^ knockout cortex.

### Sad kinases are restricted to axons and the CP

**Fig 5:**
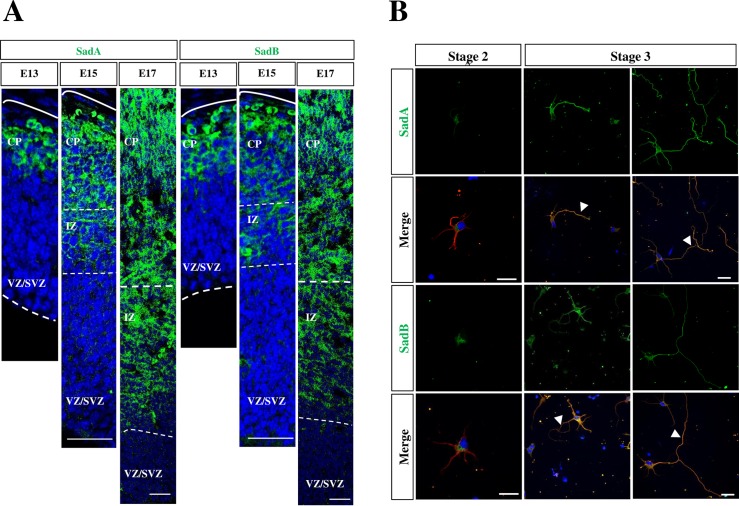
SadA and SadB can be detected in the CP and axons but not the VZ. (A) Coronal sections from the brains of E13, E15 or E17 mouse embryos were stained with Hoechst 33342 (blue) and an anti-SadA (green) antibody directed against the N-terminal kinase domain or an anti-Sad B antibody (green) directed against the C-terminus. (B) Cortical neurons from E18 rat embryos were analyzed at 1, 2 and 3 days in vitro (d.i.v., stage 2 to 3) by staining with anti-SadA (A, green), anti-SadB (A, green) and the Tuj1 (red) antibodies. Nuclei were stained with Hoechst 33342 (blue). Early and late stage 3 neurons are shown. Axons are marked by arrowheads. The scale bars are 20 μm (A) and 50 μm, respectively (B).

**Fig 6:**
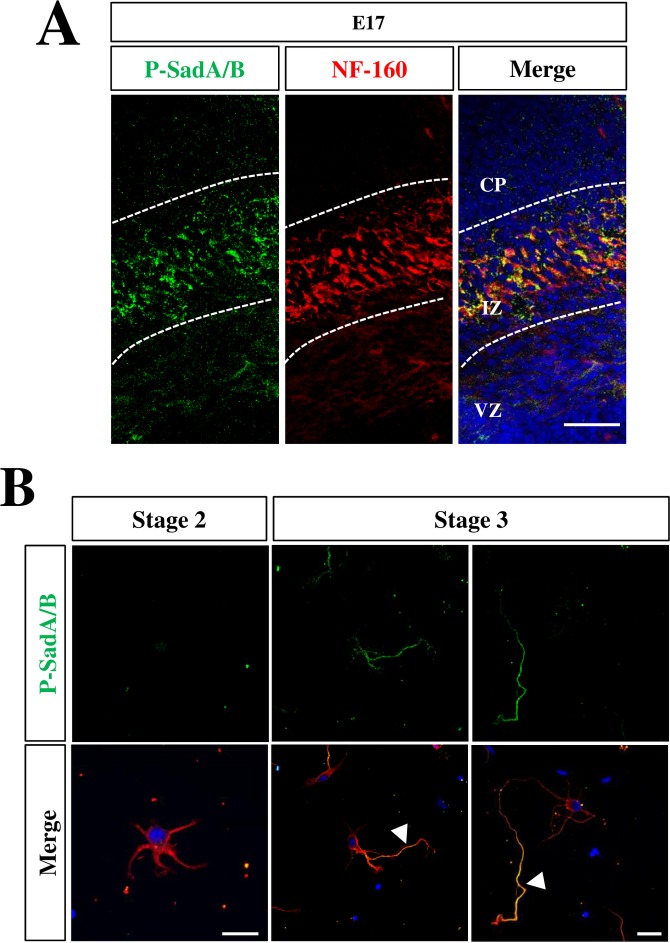
Active SadA and SadB are restricted to the axons of polarized neurons. (A) Coronal sections from the brains of E17 mouse embryos were stained with Hoechst 33342 (blue) an antibody detecting active SadA and SadB phosphorylated at Thr175 and Thr187, respectively, (P-SadA/B, green) and the Tuj1 (red) antibody. (B) Cortical neurons from E18 rat embryos were analyzed at 1, 2 and 3 days in vitro (d.i.v., stage 2 to 3) by staining Hoechst 33342 (blue) an antibody detecting active SadA and SadB phosphorylated at Thr175 and Thr187, respectively, (P-SadA/B, green) and the Tuj1 (red) antibody. Early and late stage 3 neurons are shown. Axons are marked by arrowheads. The scale bars are 20 μm (A) and 50 μm, respectively (B).

Since the loss of Sad kinases results in a decrease in the number of neural progenitors, we investigated their expression in the cortex using antibodies directed against the N-terminal kinase domain of SadA or SadB (Fig 5A, S4A Fig) and the C-terminal domain of SadB (Fig 5A). SadA and SadB were detectable in the CP and IZ and their expression increased between from E13 to E17 when the first cortical layers are formed (Fig 5A). Surprisingly, neither SadA nor SadB were detectable in APCs or IPCs at E13, E15 or E17. Co-staining with the Tuj1 antibody for neuron-specific class III β-tubulin as a marker for neurons (S4B Fig) showed that the expression of SadA was restricted to postmitotic neurons in the CP. To better correlate the expression of Sad kinases to axon formation we analyzed cultures of cortical neurons from E18 rat embryos. Expression of both SadA and SadB was very low in unpolarized neurons at stage 2 (Fig 6A). From stage 3 onwards, the expression of SadA and SadB increased and was detectable in both minor neurites and in axons as described previously [8] (Fig 6A).

SadA and SadB are activated by Lkb1 that phosphorylates them at Thr175 and Thr187, respectively [12, 13]. To determine the localization of the active Sad kinases, we stained sections and cultured cortical neurons with a phospho-specific antibody (anti-phospho-SadA/B) that detects both SadA and SadB phosphorylated at Thr175 and Thr187, respectively. The specificity of the antibody was confirmed by staining sections from the embryonic cortex and cortical neurons isolated from *Sada*^*-/-*^*;Sadb*^*-/-*^ knockout embryos that showed a complete loss of immunoreactivity (S5 Fig). In coronal sections from the E17 cortex, signals for phospho-SadA/B was restricted specifically to the IZ and co-localized with the axonal marker NF-M (Fig 5B). However, no expression was detected in the SVZ or VZ. In cultured neurons, SadA/B phosphorylation was not detectable in any neurite at stage 2 (Fig 6B). However, phospho-SadA/B was present specifically in the longest process in polarized neurons at early stage 3. When neurons are fully polarized, phospho-SadA/B was detectable only in axons. Taken together, these results show a preferential activation of Sad kinases in the axon during development while no expression was detectable in neural progenitors. This indicates that the reduction in the number of progenitors is a cell non-autonomous consequence of the *Sada*^*-/-*^*;Sadb*^*-/-*^ knockout.

### Sad kinases are required for the survival of cortical neurons

**Fig 7:**
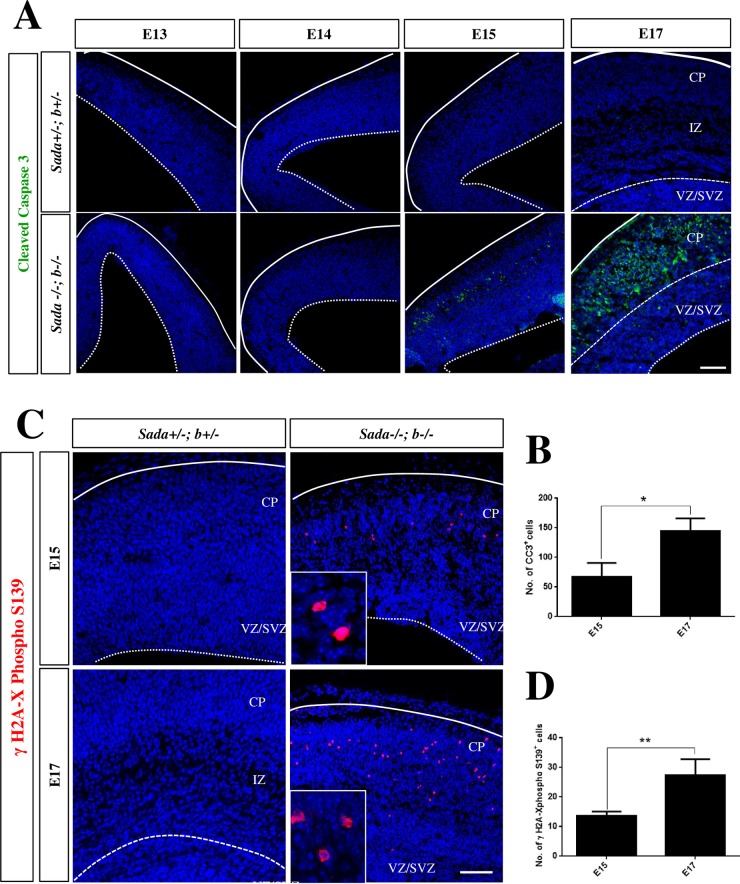
Increased apoptosis in the *Sada*^*-/-*^*;Sadb*^*-/-*^ knockout brain. (A, C) Coronal sections from the cortex of E13, E14, E15 or E17 embryos with the indicated genotypes were stained with an anti-cleaved caspase-3 (A, green) or anti-phospho-Ser139-**γ**H2A.X antibody (B, red) and Hoechst 33342 (blue). (B, D) The number of nuclei per section positive for cleaved caspase-3 (B) or phospho-S139-**γ**H2A.X (D) was determined in the cortex at the indicated stages. No signals for phospho-Ser139-**γ**H2A.X were detectable in heterozygous controls. Values are means ± s.e.m., n = 3 different embryos, Student’s t-test n between E15 and E17 (*, p<0.05; **, p<0.01). The scale bars are 50 μm.

The loss of neurons from E15 onwards could result not only from a depletion of progenitors but also from increased apoptosis. To address this possibility we stained coronal sections with an anti-cleaved caspase-3 (CC3) antibody. This analysis demonstrated the presence of a large number of CC3^+^ cells in the CP but not the VZ/SVZ of the *Sada*^*-/-*^*;Sadb*^*-/-*^ knockout brain beginning at E15 while no CC3 signals were detectable in heterozygous controls (Fig 7A). By this time early born neurons forming L5 and L6 settle in the CP. At E17, there was a striking increase in the number of CC3^+^ cells in the *Sada*^*-/-*^*;Sadb*^*-/-*^ knockout cortex (E15: 68 ± 14 cells per section, E17: 145 ± 12; Fig 7A and 7B). By contrast, no CC3^+^ cells were observed in the hippocampus or any other part of the *Sada*^*-/-*^*;Sadb*^*-/-*^ knockout brain (S6 Fig).

We also examined sections by staining for phospho-S139-**γ**H2A.X that indicates an activation of the DNA damage response pathway upon induction of DNA double strand breaks by caspase-activated DNase as a late marker for apoptosis and shows a typical pattern in apoptotic cells [24, 25]. We observed phospho-S139-**γ**H2A.X positive cells in the *Sada*^*-/-*^*;Sadb*^*-/-*^ double knockout cortex beginning at E15. This number was significantly increased at E17 (E15: 8 ± 2 cells per section in the cortex, E17: 21 ± 3; Fig 7C and 7D). By contrast, no phospho-S139-**γ**H2A.X positive cells were observed in heterozygous controls. These results show that Sad kinases are required for the survival of cortical neurons and indicate that the *Sada*^*-/-*^*;Sadb*^*-/-*^ knockout cortex shows an extensive loss of neurons due to apoptosis.

## Discussion

The analysis of the *Sada*^*-/-*^*;Sadb*^*-/-*^ knockout phenotype revealed a decrease in the number of both neural progenitors and neurons in the cortex, which coincides with an increased apoptosis from E15 onwards and is restricted to the CP but is not detectable in the VZ and SVZ. The failure to maintain the normal size of the progenitor pool is a non-cell autonomous defect since only neurons but not progenitors express Sad kinases. By contrast the hippocampal region remains unaffected and no increase in apoptosis or defects in the generation of neurons were observed.

A prominent feature of the *Sada*^*-/-*^*;Sadb*^*-/-*^ knockout is the extensive apoptosis in the CP. A similar phenotype was also observed in knockout mice for *Lkb1* and *Strada*;*Stradb*, which encode factors that activate Sad kinases [13, 16]. Previously, it was demonstrated that Sad kinases negatively regulate the cell cycle checkpoint kinase Wee1 by phosphorylating Ser642 [17]. Wee1 is expressed not only by proliferating cells but also by postmitotic neurons until stage 3 after which it is down-regulated. The phosphorylation of Ser642 and Ser123 is reduced in the brain of *Sada*^*-/-*^*;Sadb*^*-/-*^ knockout embryos suggesting that Wee1 activity is increased in the *Sada*^*-/-*^*;Sadb*^*-/-*^ knockout cortex [17, 26]. Differentiated neurons irreversibly leave the cell cycle when they become postmitotic [27]. In pathological situations like neurodegenerative diseases or after DNA damage neurons can re-enter the cell cycle but undergo apoptosis instead of dividing [28–33]. The inability to inhibit Wee1 in the *Sada*^*-/-*^*;Sadb*^*-/-*^ knockout cortex could result in a failure to maintain the postmitotic state with the consequence that the neurons undergo apoptosis.

Since Sad kinases are not expressed in neural progenitors the decreased size of the progenitor pool probably is a consequence of the loss of neurons by apoptosis. The proportion of progenitors that undergo mitosis depends on feedback signals from postmitotic neurons that regulate the cell cycle and thereby the size of the progenitor pool [34–37]. The loss of postmitotic neurons by apoptosis would reduce these feedback signals resulting in an increase in the production of neurons. Progenitor cells respond to different extracellular signals that are produced by postmitotic neurons [34–38]. These include neurotrophin-3 (NT-3), Wnt proteins and Notch signaling. The knockout of *Celsr3* and *Fzd3* e.g. causes a decrease in APCs and IPCs and increases neurogenesis due to a disrupted feedback signaling of immature neurons to APCs through the Notch pathway [35]. Down-regulation of signals like NT-3 increases the number L6 neurons due to an increased number of neurogenic divisions by progenitor cells [34, 37]. An increase in neurogenic divisions may also explain the transient increase in Ctip2^+^ cells. This possibility is supported by the observation that the mitotic index is higher in the knockout compared to the heterozygous cortex, which is indicative of a prolonged M phase in progenitors while the proportion of cell in S-phase is not changed. An increased duration of the cell cycle was shown to promote the production of neurons by symmetric neurogenic divisions that deplete the progenitor pool [39].

Taken together, the analysis of the *Sada*^*-/-*^*;Sadb*^*-/-*^ knockout shows that the Sad kinases are essential not only for neuronal polarization but also for the survival of neurons. The reduced size of the CP results from the combined effects of the loss of neurons and a depletion of progenitors. In addition, some neurons are located at deeper positions of the CP than in controls indicating a defect in neuronal migration. This could result from a failure to establish neuronal polarity as observed in cultured neurons. The loss of axons in the *Sada*^*-/-*^*;Sadb*^*-/-*^ knockout cortex could result primarily from a defect in neuronal polarization but may also be a consequence of apoptosis. Future experiments will have to address how Sad kinases promote neuronal survival and how this function is related to their role in neuronal polarity.

## Materials and methods

### Mice

*Sada*^*+/*-^ and *Sadb*^*+/-*^ mice were generously provided by Dr. Joshua Sanes (Harvard University). Genotyping was performed using the following primers: 5’- TGCCAAGTTC TAATTCCATC AGAAGCTG-3’ combined with 5’- TGCCCCTGCTCACCTTAGGTGTCACCATG -3’ and 5’- TGGGAAGGTA AGCAGGGAGG CCAGGTAACC -3’ for *Sada* and combined with 5’-TGTCTCCTAT ACCTTGATAG GTAGGCAG -3’ and 5’-AATGAAGATGG CTTGATAGGC TTACCAC -3’ for *Sadb*. Mice were housed at four to five per cage with a 12 h light/dark cycle (lights on from 07:00 to 19:00 h) at constant temperature (23°C) with *ad libitum* access to food and water. All animal protocols were carried out in accordance with the relevant guidelines and regulations and approved by the Landesamt für Natur, Umwelt und Verbraucherschutz Nordrhein-Westfalen.

### Cell culture

Cultures of hippocampal and cortical neurons were prepared from E17 mouse or E18 rat embryos as described before [40]. Dissociated neurons were plated at a density as 40,000 cells per well of a 24-well plate containing cover slips coated with poly-L-ornithine (15μg/ml, SigmaAldrich). Neurons were cultured at 37°C and 5% CO_2_ for 3 days in Neurobasal medium (Invitrogen) with supplements.

### Immunofluorescence

Brains were isolated from embryos, fixed in 4% PFA in PBS and cryoprotected in 20% sucrose/phosphate buffered saline (PBS) overnight at 4°C. After one wash in PBS, the brains were then embedded in O.C.T. medium (Tissue-Tek) and frozen on dry ice. Alternatively, E17 brains were fixed with Carnoy’s reagent, dehydrated in xylene and embedded in paraffin. Coronal 12 μm sections were cut using a cryostat or microtome (Leica). Antigen-retrieval was performed by boiling sections in 10 mM sodium citrate buffer (with 0.05% Tween20), pH 6.0 in a microwave for 10 min at 650 W The sections were blocked with 1.5% normal goat serum (PAN Biotech) in PBS with 0.03% Triton X-100 for 1 h and stained with primary antibody overnight at 4°C, and secondary antibodies in blocking buffer for 2 h at room temperature. Sections were imaged using a Zeiss 700 confocal laser-scanning microscope and single planes are displayed.

Neurons were fixed with 4% paraformaldehyde/15% sucrose in PBS for 15 min at room temperature and permeabilized with 0.1% Triton X-100/0.1% Na-Citrate/PBS for 10 min on ice. Cells were incubated with 10% normal goat serum (PAN Biotech) in PBS for 1h at room temperature, stained with primary antibody overnight at 4°C, secondary antibody for 90 min at room temperature and mounted using Mowiol (SigmaAldrich). Neuronal morphology and axon formation were analyzed as described before using a Zeiss Axiophot microscope equipped with a Visitron CCD camera and the SPOT Advanced Imaging software or a Zeiss LSM 700 using the ZEN (black edition) software [40, 41]. Image analysis was done using ImageJ 1.45s (NIH), ZEN (black edition) and Adobe Photoshop CS5.

### Antibodies

The following antibodies were used for immunofluorescence staining: rabbit anti-Brsk1 (C-terminal epitope; Cell Signaling #5935, 1:100), rabbit anti-Brsk1 (N-terminal epitope; Atlas Antibodies, HPA061719, 1:200), rabbit anti-Brsk2 (N-terminal epitope; Cell Signaling #5460, 1:100), rabbit anti-phospho-SadA/B (1:500), rabbit anti-cleaved caspase-3 (Cell Signaling, #9661, 1:200), rabbit anti-phospho-Ser139-**γ**H2A.X (Abcam, #ab11174, 1:100), mouse anti-Ki67 (Cell Signaling, #9449, 1:300), rabbit anti-Pax6 (Biolegend, #901301, 1:200), mouse anti-PH3 (Cell Signaling, #9706,1:300), mouse anti-PCNA (Millipore # MAB424), mouse anti-nestin (BD Biosciences, #611658, 1:200), rabbit anti-NF medium chain (Abcam, #ab64300, 1:200), mouse anti-NF medium chain (2H3, DSHB, 1:4), rabbit anti-Tbr1 (Abcam, #31940, 1:400), rat anti-Ctip2 (Abcam, #ab18465), SMI-312 (Abcam, #24574, 1:1000), rabbit anti-Tbr2 (Abcam, #23345, 1:400), mouse Tau-1 (Chemicon, #MAB3420; 1:500), mouse anti-MAP2 (Chemicon, #AB5622; 1:1000) and goat secondary antibodies labeled with Alexa 488 or 594 (Molecular Probes, 1:800). The rabbit anti-phospho-Sad antibody was raised against the peptide KGDSLLEpTSCGSPHY and affinity purified (Davids Biotechnology). The specificity was confirmed by staining neurons from *Sada*^*-/-*^*;Sadb*^*-/-*^ double knockout embryos, which did not show immunoreactivity [17]. Nuclei were stained with Hoechst 33342 (Molecular probes, 1:6000).

### Hematoxylin-eosin staining

Brains from E17 embryos were fixed with Carnoy’s reagent, dehydrated in xylene followed by paraffin embedding. Sections (12 μm) were deparaffinized, rehydrated and stained with Hematoxylin and Eosin using standard procedures.

### Statistical analysis

The number of cells positive for the analyzed markers was determined in a column of the CP (Ctip2, Tbr1; area delineated by the IZ/CP boundary and the MZ (control) or the SVZ/CP boundary and the MZ since IZ and CP (homozygous knockout) are not distinguishable in mutants) or VZ/SVZ (Ki67, Pax6, PCNA, PH3, Tbr2). The number of cells was normalized as the number of cells per 10^4^ μm^2^ as previously described [42]. To determine the mitotic index as the ratio of PH3^+^ and Ki67^+^cells the total number of PH3^+^ and Ki67^+^ cells per section was counted in the cortex. The number of S-phase cells was determined as the number of cells with a punctate PCNA immunoreactivity in the nucleus as described previously [23]. The total number of cells per section positive for cleaved caspase-3 (early event in apoptosis) or phospho-Ser139-**γ**H2A.X (late event in apoptosis) was quantified in the cortex (area from the cortical hem to the pallial–subpallial boundary) to investigate apoptosis. All values are means ± s.e.m. from at least three independent experiments. Statistical significance was determined using either Student’s t test or two-way ANNOVA with Sidak’s multiple comparison test (GraphPad Software and Microsoft Excel 2010) (*, p ≤0.05, **, p≤ 0.01, or ***, p≤ 0.001, ****, p≤0.0001).

## Supporting information

S1 Fig Reduced cortical size and loss of axons in *Sada*^*-/-*^*;Sadb*^*-/-*^ embryos. (A) Coronal sections from the brains of *Sada*^*+/-*^*;Sadb*^*+*/-^ and *Sada*^*-/-*^*;Sadb*^*-/-*^ E17 embryos were analyzed by Hematoxylin-Eosin staining. The scale bar is 100 μm. (B) Coronal sections from the brains of E13—E15 embryos with the indicated genotype were stained with an anti-NF-M (NF-160) antibody and Hoechst 33342 (blue). The scale bar is 50 μm.
(TIF)

S2 Fig Loss of neurofilament staining in cortical *Sada*^*-/-*^*;Sadb*^*-/-*^ neurons. Cortical (A) and hippocampal neurons (B) from E17 mouse embryos with the indicated genotypes were stained at 3 d.i.v. with anti-MAP2 (red) and anti-NF-M (green) antibodies. (C) The percentage of neurons without NF-M staining (0, red), with a single NF-M^+^ process (1, green), or with multiple NF-M^+^ processes (>1, blue) was quantified (values are means + s.e.m., n = 3 independent cultures, 100 neurons per culture, **** p<0.0001 compared to wild type, two-way ANOVA). Scale bars are 20μm. (D) Hippocampal neurons from *Sada*^*-/-*^*;Sadb*^*-/-*^ and *Sada*^*+/-*^*;Sadb*^*+*/-^ (control) E17 embryos were cultured and stained with an anti-MAP2 and the Tau-1 antibody. Hippocampal neurons from the *Sada*^*-/-*^*;Sadb*^*-/-*^ knockout extend multiple neurites that are positive for both axonal and dendritic markers. The scale bar is 20 μm.
(TIF)

S3 Fig The organization of RGCs is not affected in the *Sada*^*-/-*^*;Sadb*^*-/-*^ cortex. Coronal sections from the cortex of E13 or E17 embryos with the indicated genotypes were stained with an anti-nestin antibody. Scale bars are 100 μm.
(TIF)

S4 Fig Expression of SadA and SadB is restricted to neurons. (A) Coronal sections from the brains of E17 mouse embryos with the indicated genotypes were stained with Hoechst 33342 (blue) and an anti-SadB (green) antibody directed against the N-terminal kinase domain. (B) Coronal sections from the cortex of E15 embryos were stained with (A) anti-SadA (green) and the Tuj1 antibody (neurons, red). The scale bar is 50 μm.
(TIF)

S5 Fig Staining with the anti-phospho-Sad antibody is lost in the *Sada*^*-/-*^*;Sadb*^*-/-*^ knockout. (A) Coronal sections from the brains of E14 mouse embryos with the indicated genotypes were stained with Hoechst 33342 (blue), the SMI-312 anti-neurofilament antibody (red) as axonal marker and an antibody detecting active SadA and SadB phosphorylated at Thr175 and Thr187, respectively (P-SadA/B, green). (B) Hippocampal neurons from heterozygous *Sada*^*+/-*^*;Sadb*^*+/-*^ and homozygous *Sada*^*-/-*^*;Sadb*^*-/-*^ E17 embryos were stained with anti-MAP2 (dendritic marker, green) and anti-phospho-Sad (red) antibodies. Nuclei were stained with Hoechst 33342 (blue). The scale bar is 20 μm. The scale bars are 20 μm (A) and 50 μm (B), respectively.
(TIF)

S6 Fig The hippocampus of *Sada*^*-/-*^*;Sadb*^*-/-*^ knockout embryos does not show a reduction of progenitors or increased neuronal apoptosis. (A) Coronal sections from the hippocampus of E17 embryos with the indicated genotypes were analyzed by staining with anti-Pax6 (pseudo-colored cyan), anti-Tbr2 (green) or anti-cleaved caspase-3 (green) antibodies and Hoechst 33342 (blue). (B) The number of Pax6^+^ and Tbr2^+^ cells per 10^4^ μm^2^ was quantified in the hippocampus of *Sada*^*+/-*^*;Sadb*^*+/-*^ and *Sada*^*-/-*^*;Sadb*^*-/-*^ embryos. No significant difference was observed. Scale bars are 50 μm.
(TIF)
