# Supplementary figures and images for: The loss of the kinases SadA and SadB results in early neuronal apoptosis and a reduced number of progenitors
Source: PLoS One. 2018 Apr 26;13(4):e0196698. doi: 10.1371/journal.pone.0196698 (PMC5919486; doi:10.1371/journal.pone.0196698)

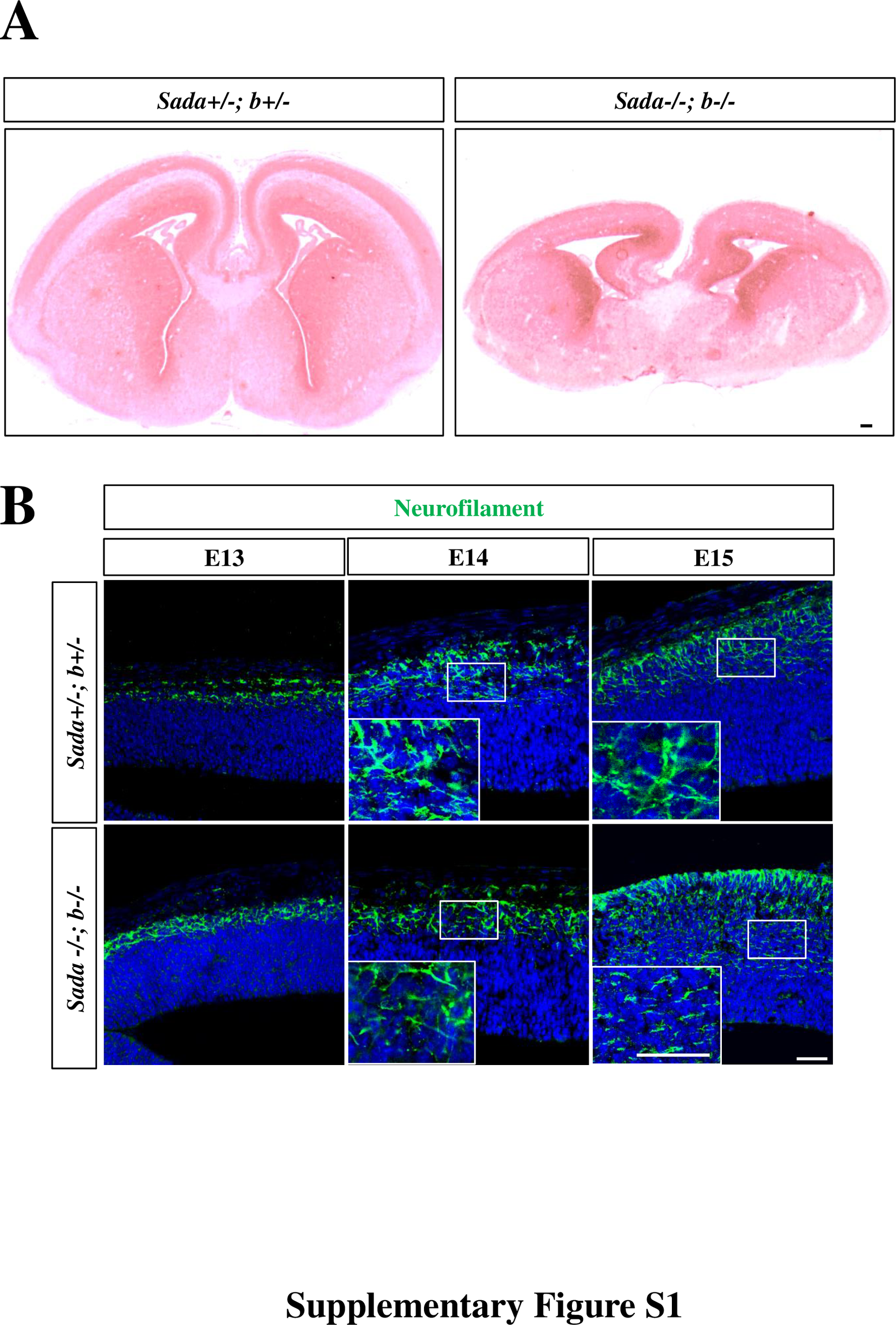

Supplement: S1 Fig — (A) Coronal sections from the brains of Sada+/-;Sadb+/- and Sada-/-;Sadb-/- E17 embryos were analyzed by Hematoxylin-Eosin staining. The scale bar is 100 μm. (B) Coronal sections from the brains of E13—E15 embryos with the indicated genotype were stained with an anti-NF-M (NF-160) antibody and Hoechst 33342 (blue). The scale bar is 50 μm. (TIF) [file pone.0196698.s001.tif]

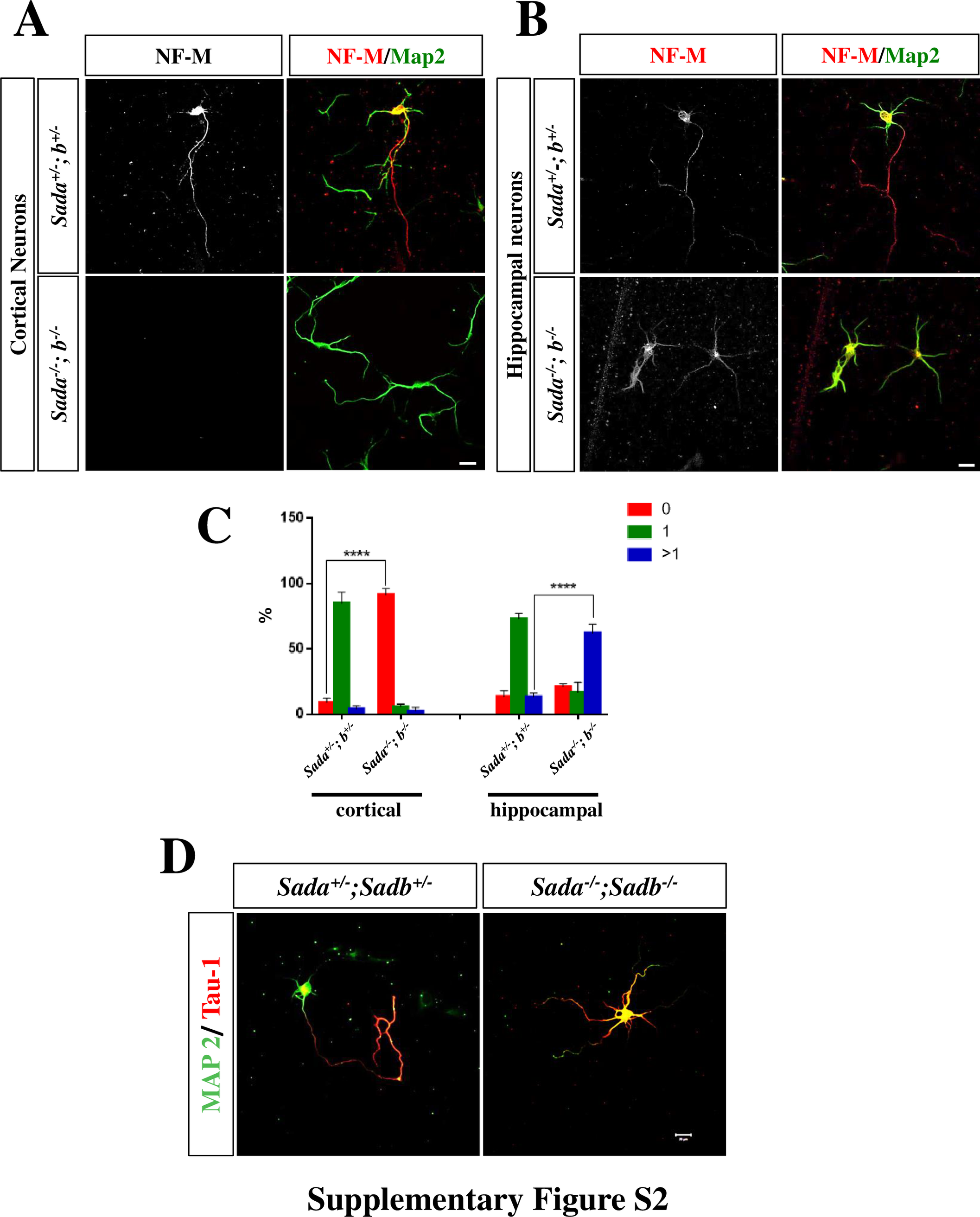

Supplement: S2 Fig — Cortical (A) and hippocampal neurons (B) from E17 mouse embryos with the indicated genotypes were stained at 3 d.i.v. with anti-MAP2 (red) and anti-NF-M (green) antibodies. (C) The percentage of neurons without NF-M staining (0, red), with a single NF-M+ process (1, green), or with multiple NF-M+ processes (>1, blue) was quantified (values are means + s.e.m., n = 3 independent cultures, 100 neurons per culture, **** p<0.0001 compared to wild type, two-way ANOVA). Scale bars are 20μm. (D) Hippocampal neurons from Sada-/-;Sadb-/- and Sada+/-;Sadb+/- (control) E17 embryos were cultured and stained with an anti-MAP2 and the Tau-1 antibody. Hippocampal neurons from the Sada-/-;Sadb-/- knockout extend multiple neurites that are positive for both axonal and dendritic markers. The scale bar is 20 μm. (TIF) [file pone.0196698.s002.tif]

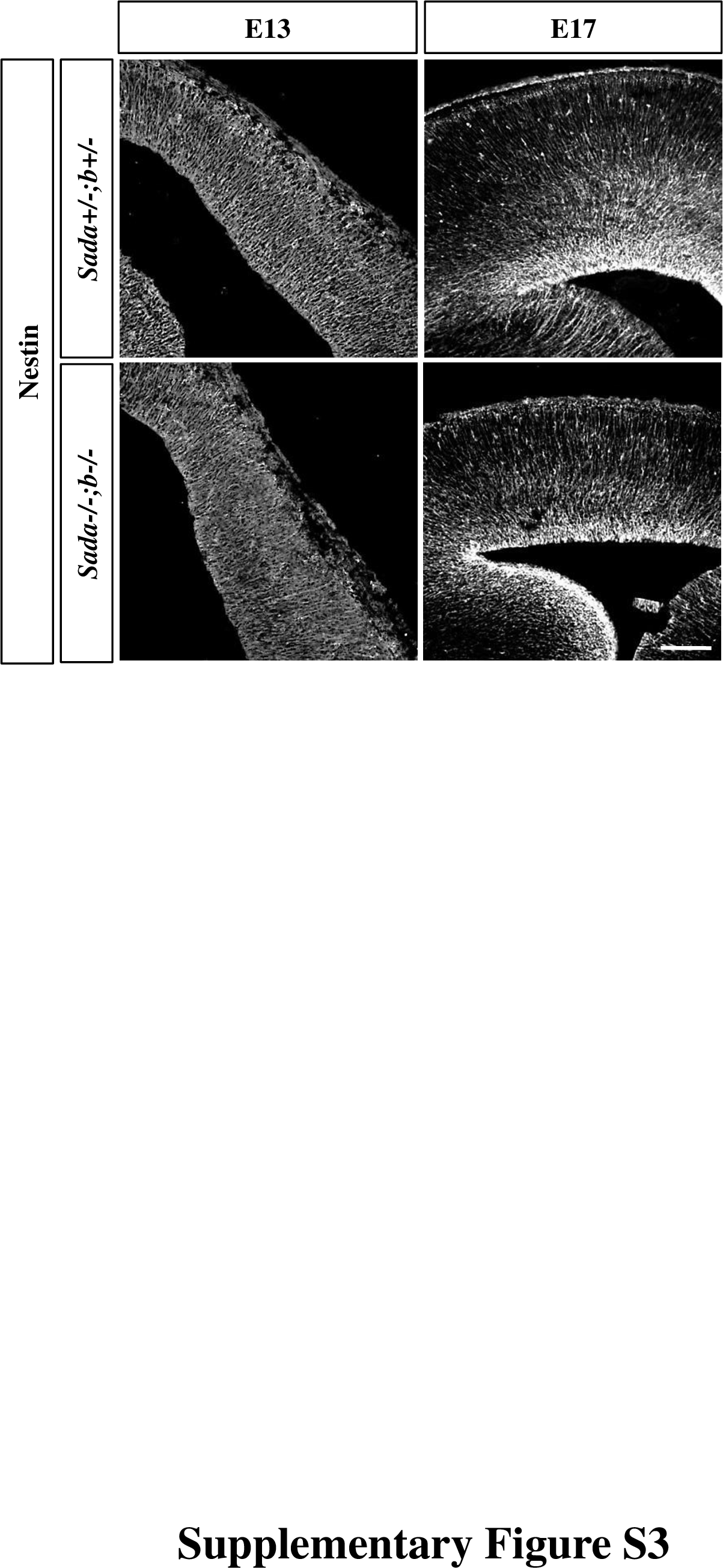

Supplement: S3 Fig — Coronal sections from the cortex of E13 or E17 embryos with the indicated genotypes were stained with an anti-nestin antibody. Scale bars are 100 μm. (TIF) [file pone.0196698.s003.tif]

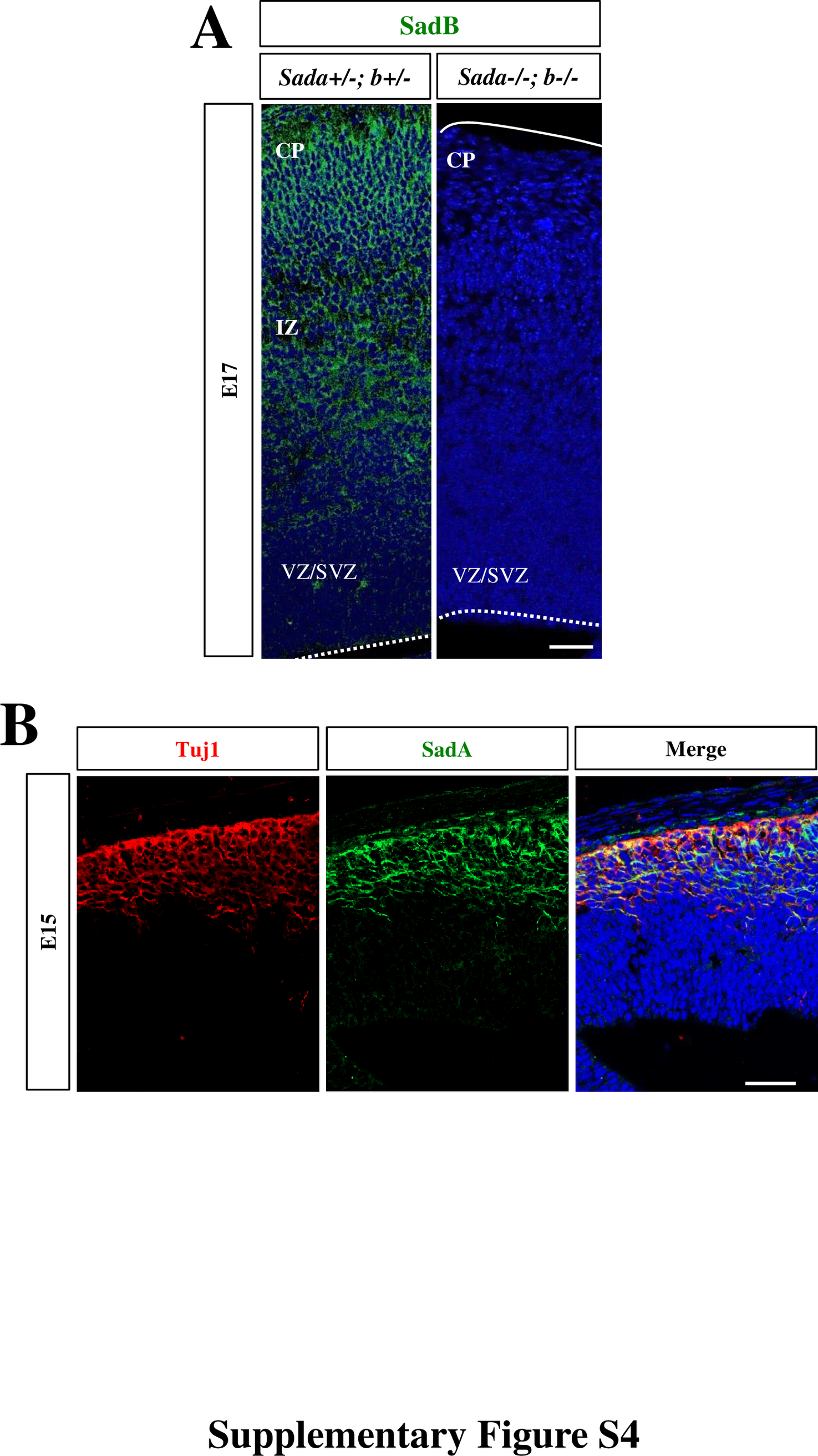

Supplement: S4 Fig — (A) Coronal sections from the brains of E17 mouse embryos with the indicated genotypes were stained with Hoechst 33342 (blue) and an anti-SadB (green) antibody directed against the N-terminal kinase domain. (B) Coronal sections from the cortex of E15 embryos were stained with (A) anti-SadA (green) and the Tuj1 antibody (neurons, red). The scale bar is 50 μm. (TIF) [file pone.0196698.s004.tif]

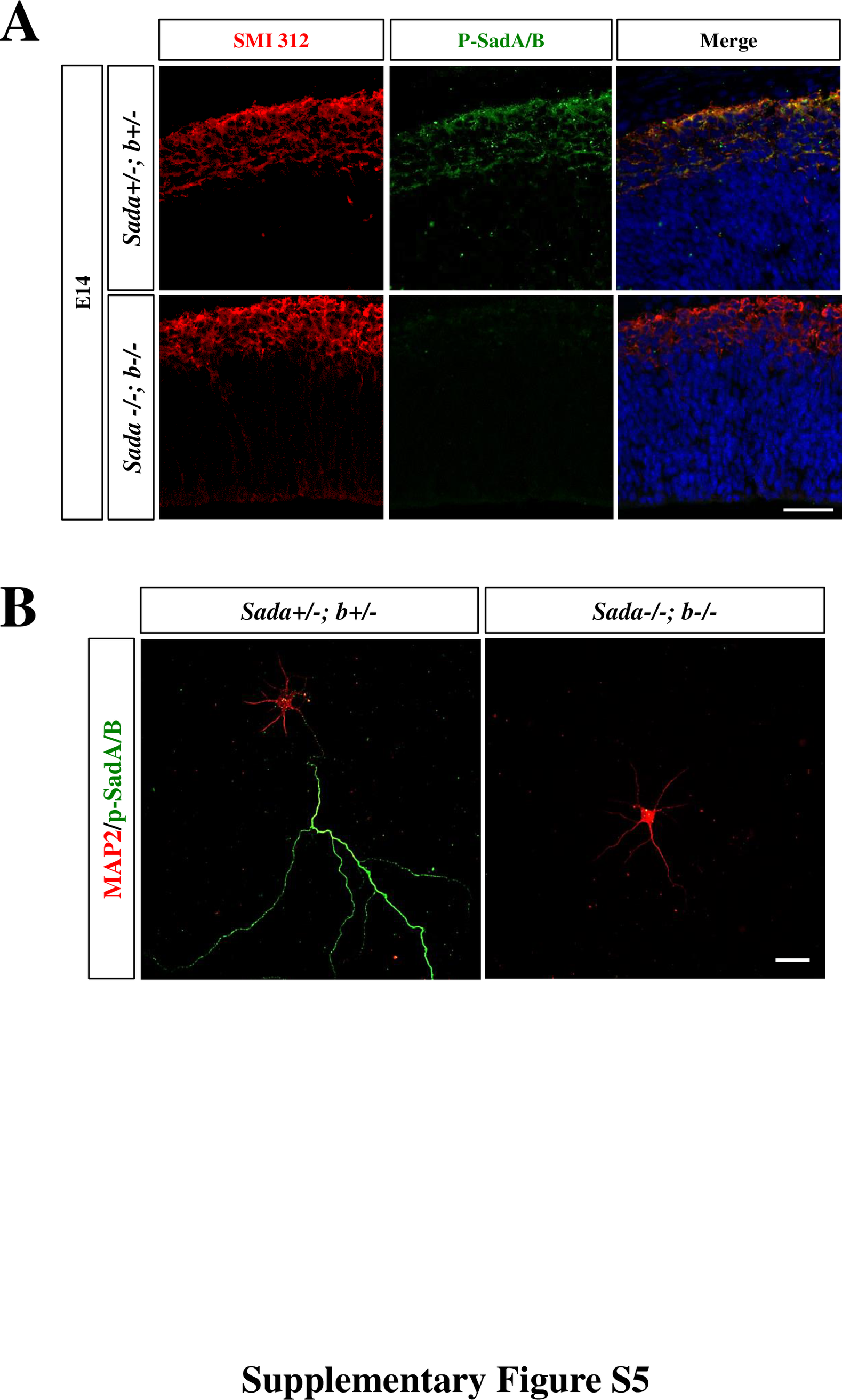

Supplement: S5 Fig — (A) Coronal sections from the brains of E14 mouse embryos with the indicated genotypes were stained with Hoechst 33342 (blue), the SMI-312 anti-neurofilament antibody (red) as axonal marker and an antibody detecting active SadA and SadB phosphorylated at Thr175 and Thr187, respectively (P-SadA/B, green). (B) Hippocampal neurons from heterozygous Sada+/-;Sadb+/- and homozygous Sada-/-;Sadb-/- E17 embryos were stained with anti-MAP2 (dendritic marker, green) and anti-phospho-Sad (red) antibodies. Nuclei were stained with Hoechst 33342 (blue). The scale bar is 20 μm. The scale bars are 20 μm (A) and 50 μm (B), respectively. (TIF) [file pone.0196698.s005.tif]

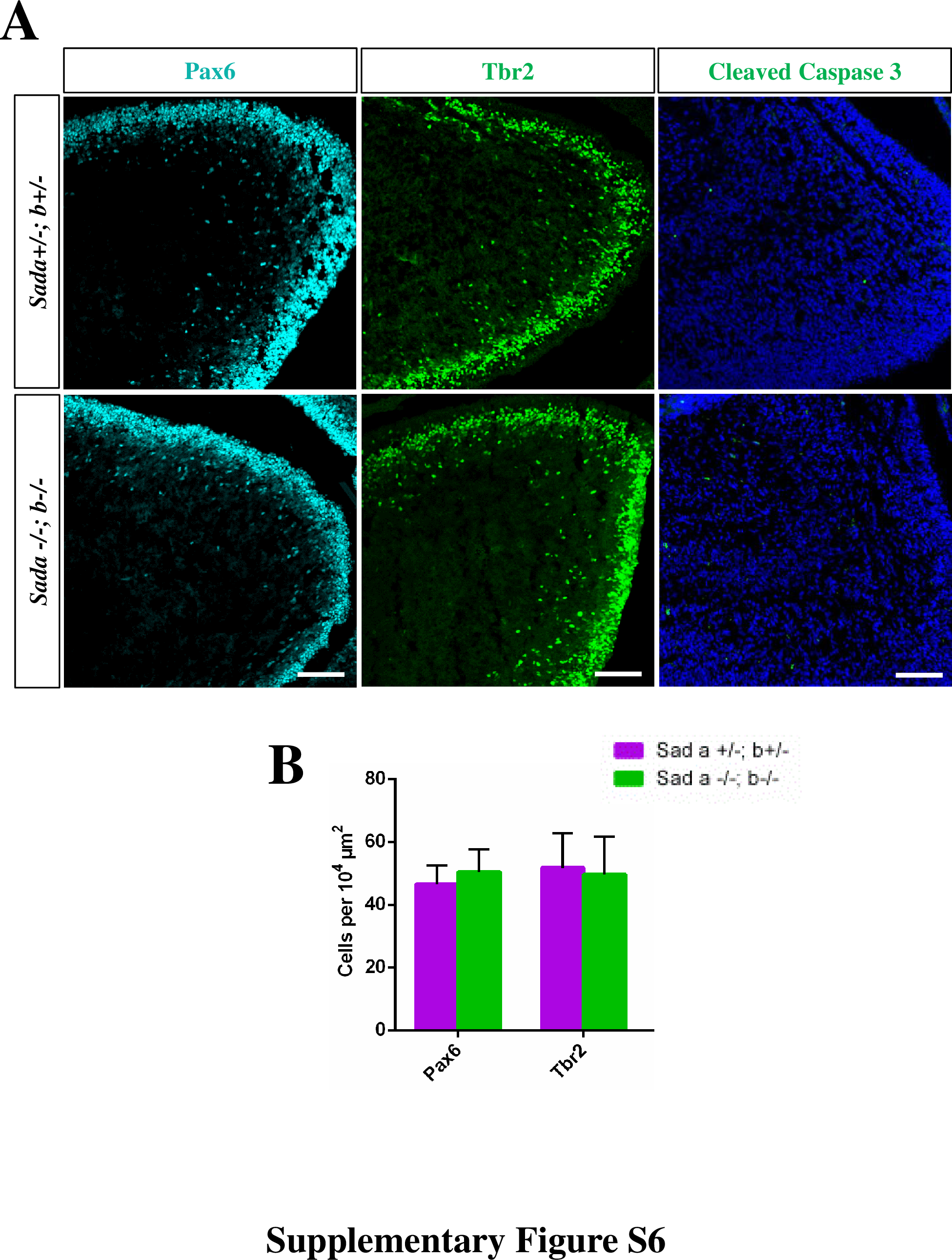

Supplement: S6 Fig — (A) Coronal sections from the hippocampus of E17 embryos with the indicated genotypes were analyzed by staining with anti-Pax6 (pseudo-colored cyan), anti-Tbr2 (green) or anti-cleaved caspase-3 (green) antibodies and Hoechst 33342 (blue). (B) The number of Pax6+ and Tbr2+ cells per 104 μm2 was quantified in the hippocampus of Sada+/-;Sadb+/- and Sada-/-;Sadb-/- embryos. No significant difference was observed. Scale bars are 50 μm. (TIF) [file pone.0196698.s006.tif]
